# A Health Disparities Perspective on Obesity Research

**Published:** 2009-06-15

**Authors:** Paula Braveman

**Affiliations:** University of California, San Francisco — Family & Community Medicine

## Abstract

Obesity is a major risk factor for chronic disease and can decrease longevity, quality of life, and economic productivity. Compelling ethical, human rights, and practical reasons exist for addressing social disparities in obesity, which requires systematically applying a disparities perspective to obesity research and relevant policy. A disparities perspective guides us to consider multiple dimensions and levels of social advantage and disadvantage and how those advantages and disadvantages produce disparities in obesity and its consequences.

## Introduction

Obesity is a major risk factor for chronic disease and can decrease longevity, quality of life, and economic productivity (1), and research should examine how obesity and its consequences are patterned socially. A health disparities perspective, which systematically examines how health is distributed across racial/ethnic and socioeconomic groups, can contribute to obesity research.

The most elegant definition of health disparities was offered by Margaret Whitehead: "Differences which are unnecessary and avoidable but, in addition, are also considered unfair and unjust" (2). The following definition is more complex and less elegant but addresses some conceptual and measurement challenges: a health disparity is a particular type of difference in health (or in the determinants of health that could be shaped by policies) in which disadvantaged social groups systematically experience worse health or more health risks than do more advantaged social groups. Disadvantaged social groups include racial/ethnic minorities, low-income people, women, or others who have persistently experienced discrimination (3). Health disparities put socially disadvantaged groups at further disadvantage regarding their health because poor health then elevates their risk of further social disadvantage (eg, through health-related job loss), which then can exacerbate their ill health, and so on (3). This compounding of disadvantage is what makes health disparities particularly unfair.

## Applying a Disparities Lens to Research on Obesity

To apply a disparities lens to any health research endeavor is to systematically seek to identify and understand disparities in health among more and less advantaged social groups. This approach contrasts with prevailing approaches to health research in the United States, which often examine racial or ethnic groups without 1) explaining why they are examined, 2) considering social class, or 3) examining race-related social factors such as racial segregation, which could strongly affect a person's health (4). If social class is considered, prevailing approaches often measure it inadequately and control for it rather than study its effects. A disparities lens highlights health or health-related differences closely linked with differences in social advantage on both socioeconomic and racial/ethnic lines. In the case of obesity research, modifiable conditions in people's lives can be examined to discover how — in homes, neighborhoods, schools, and workplaces — they affect the likelihood of attaining and maintaining healthy weight.

**Figure 1:**
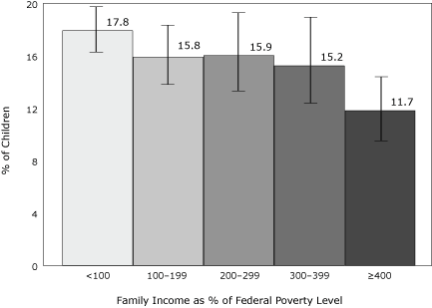
Prevalence (95% confidence interval) of obesity among children (2-2-19 years, age adjusted), according to family income as a percentage of the federal poverty level; the federal poverty level during 2004 was $18,850 for a family of 4 (7). Data source: National Health and Nutrition Examination Survey, 1999-2004.

The stepwise incremental socioeconomic differences observed in many health outcomes, whereby health gradually improves as socioeconomic factors improve (5), are not as clear-cut for obesity. For example, obesity among women followed a marked socioeconomic gradient during the 1970s, but more recently the socioeconomic disparities in obesity have actually narrowed because of a larger increase in obesity among higher than among lower socioeconomic status groups (6). Among children, obesity appears to be related to family income but does show a clear stepwise pattern (Figure 1). The poorest have the highest and the richest the lowest obesity rates; the middle groups, however, appear similar to one other. Adult obesity prevalence has a similar pattern.

**Table:** 

**Family Income, % of Federal Poverty Level**	**% of Children (95% Confidence Interval)**
<100	17.8 (16.0-19.7)
100–199	15.8 (13.6-18.2)
200–299	15.9 (13.1-19.1)
300–399	15.2 (12.2-18.7)
≥400	11.7 (9.5-14.3)

To complicate matters, patterns of obesity — and of socioeconomic disparities in obesity — vary markedly by race or ethnic group, by sex, and over time (8). This complexity presents an opportunity to obtain clues about potentially remediable causes. Patterns that shift across social groups over time suggest that modifiable factors are probably involved.

### Ethical and human rights reasons

Ethical principles dictate that all people should be valued equally and have equal opportunities to be healthy. Health is essential for well-being and economic self-sufficiency; thus, resources needed for health should be distributed equitably, which many have interpreted to mean according to need. It is difficult, however, to define and measure need (3,9,10).

Human rights principles can provide assistance. According to well-established human rights norms and agreements signed by almost all nations, the right to health is the right to attain the highest level of health possible (11-13). This concept has been criticized for being difficult to operationalize for measurement purposes (14). However, the health of the most privileged social groups (eg, the dominant racial/ethnic group or the wealthiest group) indicates what should be possible for everyone (3,15). Human rights norms, principles, and agreements oblige governments to make good faith efforts toward progressively removing obstacles to all people's realizing their full health potential, particularly for those social groups that historically have experienced more obstacles.

### Practical reasons

Differences in a range of health indicators according to diverse measures of social class have been observed for centuries (16) and across virtually all societies in which they have been studied (17). Health has been reported by race in the United States for more than a century (18,19), although the issue of racial/ethnic disparities in health became prominent only during the past 2 decades (20,21). Many health researchers routinely report results by racial/ethnic group but do not examine health differences by markers of social class, such as income or education. Inadequate socioeconomic information often has reinforced a widespread tendency to make unfounded assumptions about the nature of racial or ethnic disparities in health, reifying genetic or "cultural" explanations and deflecting attention from potentially remediable social factors (22).

A disparities lens can focus on neglected factors in health research, increasing the likelihood of sound conclusions, not only about social disparities in health but also about a range of research questions, including ones assumed to be purely biomedical. Failure to adequately consider social factors can result in erroneous conclusions from research findings, even on many questions that are not in themselves focused on social issues but for which social factors may play a role as confounders, mediators, or effect-modifiers (23).

## Neglected Dimensions to Consider in Applying a Disparities Lens

The human rights concept of removing obstacles to realizing rights, including the right to health, particularly among those who historically have experienced more obstacles, can enrich obesity research. It can push us to focus on the root causes of social advantage and disadvantage implicated in obesity disparities. This approach contrasts with the prevailing approach, which generally focuses on the behaviors that immediately lead to obesity without considering the factors that shape the behaviors.

Examination of social disadvantage and advantage is crucial to social disparities research. It is challenging, however, because of the limited amount of information on them in most health studies. Social advantage or disadvantage can be material, psychosocial, or both.

### Material and psychosocial dimensions of social advantage and disadvantage

Social disadvantage can be based on material conditions, determined by access to resources and services that affect health such as adequate nutrition, sanitation, housing, and medical care. It also can be of a psychosocial nature, based on human relationships and their psychological effects. For example, unfair treatment based on one's race or ethnic group can cause psychological distress. In addition, one's awareness of being in a group that has historically suffered discrimination could act as a chronic stressor, even in the absence of overt incidents of unfair treatment. These dimensions often coexist and interact. Material disadvantage (eg, resulting from inadequate income or wealth) can affect obesity by influencing the ability to purchase nutritious food or to live in a neighborhood with safe, pleasant places to exercise and markets that sell affordable fresh produce. Material hardship also could increase obesity risk insofar as it is a source of chronic stress; stress could limit people's ability to change weight-related behaviors even when informed and motivated (24-26). Low educational attainment could increase the risk of obesity by limiting economic opportunities or one's ability to understand and act on health information.

Racial or ethnic group is closely associated with social advantage and disadvantage and with health disparities. Although each broad racial or ethnic group is heterogeneous, overall, blacks, Hispanics, and American Indians have the lowest and Asian Americans the highest incomes and educational levels; whites have intermediate levels (27-29). Racial or ethnic differences thus often reflect socioeconomic differences, which can affect health through material pathways (23).

Experiences of racism could include not only overt incidents of intentional discrimination but also experiences in which unintended harm results because of deeply rooted structural arrangements, such as those perpetuating racial residential segregation. Racial segregation systematically deprives blacks and Hispanics of opportunities to live in health-promoting neighborhoods, in part by constraining their economic opportunities (5,30). These experiences could deleteriously affect health outcomes both through material pathways and through psychosocial pathways involving stress and physiologic responses to stress related to awareness of unfair treatment or stigmatization (as a member of a socially excluded group). Recent advances in understanding the neurophysiology of stress and its effects on chronic disease have greatly increased our ability to understand how both material and psychosocial disadvantage can harm health (31).

Any condition associated with stigma or lower social acceptance — such as obesity — could lead to social disadvantage and accompanying adverse health effects that are not intrinsic to that condition. Adverse health effects could occur through material or psychosocial pathways. Examples include physical or mental disability, HIV infection, or other highly stigmatized diseases. Similarly, nonheterosexual orientation can result in discrimination or social exclusion, putting one's health at risk in multiple ways. These experiences of discrimination are rarely measured in health studies.

### Time dimension

Time is another dimension of social advantage and disadvantage that can be crucial to understanding health disparities. It seems likely that not only the depth but also the duration of exposure to disadvantage could matter greatly for health. Exposures that are potentially obesigenic, such as a high-calorie diet, a crime-infested neighborhood without safe places to exercise or play, or a resource-strapped school that offers children few opportunities for supervised exercise, will likely have a larger effect given a longer duration. Yet even when these factors are measured at all, time is rarely considered. Current or last year's income may be measured but generally not whether a person was poor as a child. This oversight tends to underestimate racial/ethnic disparities in social advantage. Our research with population-based data on postpartum women in California confirms that at each level of current income or education, non-Hispanic black and Hispanic women are more likely than their non-Hispanic white counterparts to have grown up in households of lower socioeconomic status (as reflected by their parents' educational attainment) (23).

**Figure 2:**
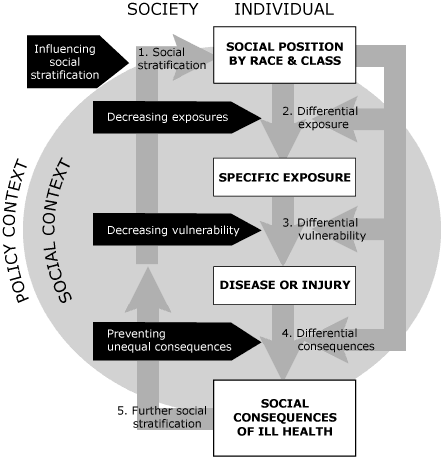
How health disparities are produced and reproduced across a lifetime and generations, and possible points to intervene. Adapted from Finn Diderichsen, Karolinska Institute, Stockholm; reprinted with permission.

A disparities perspective leads us to ask about not only the antecedents of disparities in obesity but also the differential consequences of obesity for people in different social groups. Finn Diderichsen of the Karolinska Institute in Stockholm has developed a schematic diagram highlighting the dynamic nature of how health disparities are produced and reproduced over time. Figure 2, adapted from Diderichsen, depicts how social position or stratification (the extent to which different groups are sorted into hierarchies of wealth, influence, and opportunities) leads to different health-promoting or health-damaging exposures for different social groups. Differences in social position influence not only whether a person is exposed to a given health risk but also differential vulnerability to disease incidence and severity and subsequent social consequences of illness. For example, a highly educated person who, because of obesity, develops heart disease with activity limitations may be less likely than a manual worker with little schooling to become unemployed. The highly educated person is more likely than the manual worker to have work that is knowledge-based, less affected by physical capacity, and more easily performed at home or on a more flexible schedule.

Diderichsen's diagram calls attention to multiple levels at which pathways toward health disparity can be interrupted by policies, from the most proximal level (proximal or downstream in relation to the outcome; eg, medical treatment ameliorating the health damage done by harmful exposures without addressing the exposures themselves) to the most distal level (policies in the social context that may blunt the degree of social stratification, such as policies supporting universal high-quality education beginning in early childhood, and poverty reduction).

## Levels of analysis to consider

Prevailing research approaches tend to examine 1 level of aggregation or analysis — usually the individual or household level. An accumulating body of evidence indicates, however, that characteristics of the places where we live, work, and learn may have health effects beyond the effects of factors at the individual level. For example, the health effects of being poor in a neighborhood with a high concentration of poor households could be different from the consequences of being poor in a neighborhood with a high concentration of richer households. These effects may not be simple or predictable. Being poor in a poor neighborhood may carry a higher risk of obesity, for example, if the poor neighborhood lacks accessible, affordable sources of fresh foods, safe places to exercise, or social norms that value healthy eating and exercise (32-34). Some multilevel research also has suggested that being poor in a more affluent neighborhood may have adverse psychological effects if one feels inferior to one's neighbors (35,36). Similarly, for a person of a racial/ethnic minority, if living in a neighborhood with a high concentration of people of the same racial/ethnic minority meant living under conditions that make healthy eating and exercise more difficult, obesity risks could be elevated (37). Being the lone person of a racial/ethnic minority in a neighborhood could, however, have negative psychological effects (eg, feelings of isolation, exclusion, or less sense of belonging, or experiencing overt bias), which could outweigh the positive effects of being in a place with better resources and services (35,38).

## Conclusions

A disparities lens has much to contribute to health research in general and to obesity research in particular. Focusing on disparities can guide us to examine multiple dimensions and levels of advantage or disadvantage, relative and absolute deprivation, discrimination, and social exclusion. This perspective leads us to consider conditions in both social and physical environments at the individual/household/family and the community levels that can create opportunities and resources or obstacles to health. Our attention is drawn to exposure to advantage and disadvantage over time and to determinants of vulnerability to exposure effects. We are reminded of an array of sources of advantage and disadvantage, including cumulative stress related to material poverty and the psychosocial stressors that often accompany it. Such a research framework encourages us to consider social factors that seem distal to obesity but could be highly relevant to experiences and behaviors that result in biological processes underlying obesity and its adverse clinical and social sequellae. A disparities perspective encourages us not to rely entirely on simple categorizations of social advantage or disadvantage, such as low income or educational level, but to examine the actual distributions of the relevant factors and how they relate to the health indicator of interest. Appropriate cut-points may vary across indicators; Figure 1 shows that simple dichotomies (low income vs all others) do not fit the data. A disparities perspective leads us to examine both race and social class, together and separately, as points of departure.

A disparities perspective leads us to ask the questions: What causes, exacerbates, or ameliorates racial or ethnic or socioeconomic differences in obesity during a person's lifetime and across generations? Where and how can the pathways to obesity disparities be interrupted most effectively and efficiently? Do interventions that decrease obesity prevalence at the population level also reduce obesity disparities across social groups, and vice versa? What are the differential consequences of obesity, in health and social terms, for people in different social groups? To answer these questions we must study social factors rather than attempt to control for them. High-quality disparities research looks for the root causes of social disparities in health to inform efforts to intervene. In contrast, prevailing approaches often take poverty, near-poverty, and institutionalized racial bias as givens and focus primarily on how to buffer the health-damaging effects. A disparities lens can make practical contributions to obesity research, even regarding questions whose central focus is not disparities.
